# Supplementary material for: The Evolution and Characterization of the RNA Interference Pathways in Lophotrochozoa
Source: Genome Biol Evol. 2024 May 7;16(5):evae098. doi: 10.1093/gbe/evae098 (PMC11114477; doi:10.1093/gbe/evae098)
Supplement: evae098_Supplementary_Data [file evae098_supplementary_data.zip › Supplementary_materials.zip!/selected_miRNAs/NoGe_selected_miRNAs/mirdeep_pdf_files/NMRB01000795.1_Notospermus_geniculatus_strain_Ushimado_scaffold795,_whole_genome_shotgun_sequence_20955.pdf]

Provisional ID : NMRB01000795.1_Notospermus_geniculatus_strain_Ushimado_scaffold795,_whole_genome_shotgun_sequence_20955

Score total	:	2573.7
Score for star read(s)	:	3.9
Score for read counts	:	2569.1
Score for mfe	:	1.3
Score for randfold	:	0
Score for cons. seed	:	-0.6
Total read count	:	5051
Mature read count	:	4941
Loop read count	:	0
Star read count	:	110

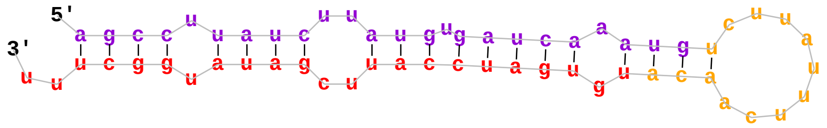

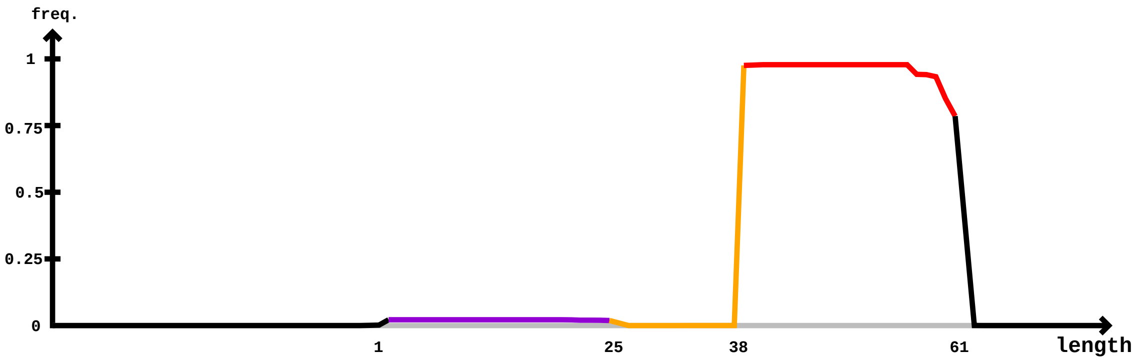

Star Mature

```
5'- guuuggucuccgacagggagggaugaguacugcagccuuaucuuaugugaucaaaugucuuauuucaacaugugauccauucgauauggcuuugguacaaacuccaaa  -3'   obs
    guuuggucuccgacagggagggaugaguacugcagccuuaucuuaugugaucaaaugucuuauuucaacaugugauccauucgauauggcuuugguacaaacuccaaa          exp
    .......((((.....))))(((...((((((.((((.((((..(((.(((((.((((.........)))).))))))))..)))).)))).))))))....)))...   reads   mm    sample
    ...............................gcagccuuaucuuaugugauc........................................................   2       0     Mm2
    ...............................gcagccuuaucuuaugugauca.......................................................   2       0     Mm2
    ................................cagccuuaucuuaugugauca.......................................................   1       0     Mm2
    ................................cagccuuaucuuaugugCucaa......................................................   1       1     Mm2
    .................................agccuuaucuuaugugaucaaau....................................................   4       0     Mm2
    .................................agccuuaucuuaugugaucaaaug...................................................   60      0     Mm2
    ................................................................ucaacaugugauccauucgauaug....................   1       0     Mm2
    ......................................................................ugugauccauucgauauC....................   1       1     Mm2
    ......................................................................ugugauccauucgauaug....................   42      0     Mm2
    ......................................................................ugugauccauucgauaugg...................   2       0     Mm2
    ......................................................................ugugauccauucgauaugU...................   2       1     Mm2
    ......................................................................ugugauccauucgauauggA..................   1       1     Mm2
    ......................................................................ugugauccauucgauauggc..................   18      0     Mm2
    ......................................................................ugugauccauucgauauCgcu.................   1       1     Mm2
    ......................................................................ugugaAccauucgauauggcu.................   1       1     Mm2
    ......................................................................ugugauccauucgauauggcu.................   160     0     Mm2
    ......................................................................ugugauccauucgauauUgcu.................   1       1     Mm2
    ......................................................................ugugauccauucgauaAggcu.................   2       1     Mm2
    ......................................................................ugugauccauucgauauggcG.................   4       1     Mm2
    ......................................................................ugugauccauucgauauggcuu................   162     0     Mm2
    ......................................................................ugugauccauucgauauCgcuu................   1       1     Mm2
    ......................................................................ugugauccauucgauaAggcuu................   1       1     Mm2
    ......................................................................ugugauccauucgauauggcuG................   1       1     Mm2
    ......................................................................ugugauccauucgauauggcCu................   1       1     Mm2
    ......................................................................uguAauccauucgauauggcuu................   2       1     Mm2
    ......................................................................ugugauccauucAauauggcuu................   1       1     Mm2
    ......................................................................ugugauccauucgauaGggcuu................   1       1     Mm2
    ......................................................................uguCauccauucgauauggcuu................   1       1     Mm2
    ......................................................................ugugauccauAcgauauggcuuu...............   5       1     Mm2
    ......................................................................ugugUuccauucgauauggcuuu...............   1       1     Mm2
    ......................................................................ugugauccaCucgauauggcuuu...............   1       1     Mm2
    ......................................................................ugugauccCuucgauauggcuuu...............   1       1     Mm2
    ......................................................................ugugauccauGcgauauggcuuu...............   2       1     Mm2
```

```
                                 Star                                 Mature
guuuggucuccgacagggagggaugaguacugcagccuuaucuuaugugaucaaaugucuuauuucaacaugugauccauucgauauggcuuugguacaaacuccaaa
......................................................................ugugaucUauucgauauggcuuu...............  1     1  Mm2
......................................................................ugugauccauucgauauUgcuuu...............  10    1  Mm2
......................................................................ugugauccauucgauauggUuuu...............  1     1  Mm2
......................................................................ugugauccauucgauaAggcuuu...............  5     1  Mm2
......................................................................ugugauccauucgauaugUcuuu...............  2     1  Mm2
......................................................................ugugauccauucAauauggcuuu...............  1     1  Mm2
......................................................................ugugauccauucgauCuggcuuu...............  1     1  Mm2
......................................................................ugugaAccauucgauauggcuuu...............  1     1  Mm2
......................................................................ugugauccauucgauaCggcuuu...............  8     1  Mm2
......................................................................ugugauccauucgauauggcuuC...............  1     1  Mm2
......................................................................ugugCuccauucgauauggcuuu...............  1     1  Mm2
......................................................................ugugauccGuucgauauggcuuu...............  1     1  Mm2
......................................................................ugCgauccauucgauauggcuuu...............  2     1  Mm2
......................................................................ugugauccauucgauauggcuAu...............  1     1  Mm2
......................................................................ugugauccauucgauauggcuuA...............  1     1  Mm2
......................................................................ugugauccUuucgauauggcuuu...............  1     1  Mm2
......................................................................ugugauccauucgauauggcuuu...............  2216  0  Mm2
......................................................................Cgugauccauucgauauggcuuu...............  3     1  Mm2
......................................................................ugugauAcauucgauauggcuuu...............  1     1  Mm2
......................................................................uguAauccauucgauauggcuuu...............  1     1  Mm2
......................................................................ugugauccauucUauauggcuuu...............  1     1  Mm2
......................................................................ugugauccauucgauauCgcuuu...............  5     1  Mm2
......................................................................ugGgauccauucgauauggcuuu...............  1     1  Mm2
......................................................................ugugauccauucgauauggcuuG...............  2     1  Mm2
......................................................................ugugauccauCcgauauggcuuu...............  1     1  Mm2
......................................................................ugugauccauucgauaGggcuuu...............  2     1  Mm2
......................................................................ugugauccauucgauauAgcuuu...............  13    1  Mm2
......................................................................ugugauccauucgauGuggcuuu...............  2     1  Mm2
......................................................................ugugauccauucgauauggcCuu...............  3     1  Mm2
......................................................................ugAgauccauucgauauggcuuu...............  1     1  Mm2
......................................................................ugugauccauucgauaugCcuuu...............  1     1  Mm2
......................................................................ugugauccauucgaGauggcuuu...............  1     1  Mm2
......................................................................ugugauccauucgauauggcuuuU..............  88    1  Mm2
......................................................................ugugauccauucgauauggcuuuC..............  1     1  Mm2
......................................................................ugugauccauucgauauggcuuuA..............  4     1  Mm2
.......................................................................gugauccauucgauauUgcuuu...............  1     1  Mm2
.......................................................................gugauccauucgauauggcuuu...............  6     0  Mm2
........................................................................ugauccauucgauauggcuuu...............  5     0  Mm2
........................................................................ugauccauucgauauggcuuugg.............  1     0  Mm2

................................cagccuuaucuuaugugauca.......................................................  3     0  Mm1
.................................agccuuaucuuaugugaucaaaug...................................................  11    0  Mm1
......................................................................ugugUuccauucgauaug....................  1     1  Mm1
......................................................................ugugauccauucgauaGg....................  2     1  Mm1
......................................................................ugugauccauucgauUug....................  1     1  Mm1
......................................................................ugugauccauucgauaug....................  110   0  Mm1
......................................................................uAugauccauucgauaug....................  1     1  Mm1
......................................................................ugugauccauucgauauU....................  1     1  Mm1
......................................................................uCugauccauucgauaug....................  1     1  Mm1
......................................................................ugugauccauucgauaAg....................  1     1  Mm1
......................................................................ugugauccauucgauauggc..................  16    0  Mm1
......................................................................ugugauccauucgauauAgcu.................  1     1  Mm1
......................................................................ugugauccauucgauaCggcu.................  1     1  Mm1
......................................................................ugugauccUuucgauauggcu.................  1     1  Mm1
......................................................................ugugauccauuUgauauggcu.................  1     1  Mm1
......................................................................ugugauccauucgauauggcu.................  174   0  Mm1
......................................................................ugugauccauucgauauggcG.................  4     1  Mm1
......................................................................ugugauccauucgauauggcC.................  1     1  Mm1
......................................................................ugugauccauucgauauCgcu.................  1     1  Mm1
......................................................................ugugauccauucgauauUgcuu................  1     1  Mm1
......................................................................ugugauccauucgauaCggcuu................  2     1  Mm1
......................................................................ugugauccauucgauauggcuu................  129   0  Mm1
......................................................................ugugauUcauucgauauggcuuu...............  2     1  Mm1
......................................................................ugugUuccauucgauauggcuuu...............  1     1  Mm1
......................................................................ugugauccGuucgauauggcuuu...............  1     1  Mm1
......................................................................ugugauccauucgauauCgcuuu...............  6     1  Mm1
......................................................................ugugauccauuUgauauggcuuu...............  1     1  Mm1
......................................................................ugugauccauGcgauauggcuuu...............  3     1  Mm1
......................................................................ugugauccauucgauaGggcuuu...............  4     1  Mm1
......................................................................ugugauccauucgauauUgcuuu...............  6     1  Mm1
```

```
                                 Star                                  Mature
guuuggucuccgacagggagggaugaguacugcagccuuaucuuaugugaucaaaugucuuauuucaacaugugauccauucgauauggcuuugguacaaacuccaaa
......................................................................ugugauccauucgauaCggcuuu...............   6     1   Mm1
......................................................................ugugauccauucgauauggcuuu...............   971   0   Mm1
......................................................................ugugauccauAcgauauggcuuu...............   3     1   Mm1
......................................................................ugugauccauucgauauggcCuu...............   1     1   Mm1
......................................................................ugugauccauucgauaAggcuuu...............   4     1   Mm1
......................................................................ugugauccauucgauauAgcuuu...............   4     1   Mm1
......................................................................ugugauccauucgauauggcuuA...............   20    1   Mm1
......................................................................Cgugauccauucgauauggcuuu...............   1     1   Mm1
......................................................................ugugauccauucgauauggcuuuU..............   8     1   Mm1
......................................................................ugugauccauucgauauggcuuug..............   2     0   Mm1
......................................................................ugugauccauucgauauggcuuuA..............   11    1   Mm1

......................................................................ugugauccUuucgauauggcuuu...............   1     1   Fm2
......................................................................ugugauccauucgauauggcuuu...............   13    0   Fm2
......................................................................ugugauccauucgauauUgcuuu...............   2     1   Fm2
......................................................................ugugauccauucgauaGggcuuu...............   1     1   Fm2

......................................................................ugugauccauucgauauggcuu................   2     0   Fm1
......................................................................ugugauccauucgauauggcuuu...............   4     0   Fm1
......................................................................ugugauccauucgauauggcuuuU..............   6     1   Fm1

.................................agccuuaucuuaugugaucaaau....................................................   1     0   Mm3
.................................agccuuaucuuaugugGucaaaug...................................................   3     1   Mm3
.................................agccuuaucuuaugugaGcaaaug...................................................   1     1   Mm3
.................................agccuuaucuuaugugaucaaaug...................................................   15    0   Mm3
.................................agccuuaucuuaugugCucaaaug...................................................   1     1   Mm3
.................................agccuuaucuuaugugaucaaaugu..................................................   2     0   Mm3
......................................................................ugugauccauucgauaug....................   19    0   Mm3
......................................................................ugugauccauucgauauA....................   1     1   Mm3
......................................................................ugugauccauucgauauU....................   1     1   Mm3
......................................................................ugugauccauucgauaugg...................   1     0   Mm3
......................................................................ugugauccauucgauauggc..................   4     0   Mm3
......................................................................ugugauccauCcgauauggc..................   1     1   Mm3
......................................................................ugugauccauucgauauggcu.................   49    0   Mm3
......................................................................ugugauccauucgauauAgcu.................   1     1   Mm3
......................................................................ugugauccauAcgauauggcu.................   1     1   Mm3
......................................................................ugugauccUuucgauauggcu.................   1     1   Mm3
......................................................................ugugauccauucgauauggcuu................   26    0   Mm3
......................................................................ugugauccauucgauauAgcuu................   1     1   Mm3
......................................................................ugugauccGuucgauauggcuuu...............   1     1   Mm3
......................................................................ugugauccauucgauauAgcuuu...............   2     1   Mm3
......................................................................ugugauccauCcgauauggcuuu...............   3     1   Mm3
......................................................................ugugauccauucgauauggcuuu...............   434   0   Mm3
......................................................................ugugauccauAcgauauggcuuu...............   2     1   Mm3
......................................................................ugugauccauucgauauggcuuA...............   4     1   Mm3
......................................................................ugugauccauucgauaCggcuuu...............   2     1   Mm3
......................................................................ugugauccauGcgauauggcuuu...............   1     1   Mm3
......................................................................ugugauccauucgauauCgcuuu...............   1     1   Mm3
......................................................................ugugauccauucgauauUgcuuu...............   2     1   Mm3
......................................................................ugugauccauucgauaAggcuuu...............   1     1   Mm3
......................................................................ugugauccUuucgauauggcuuu...............   1     1   Mm3
......................................................................ugugauccauucgauGuggcuuu...............   1     1   Mm3
......................................................................ugugauccauucgauauggcuuuA..............   3     1   Mm3
......................................................................ugugauccauucgauauggcuuuC..............   2     1   Mm3
......................................................................ugugauccauucgauauggcuuuU..............   14    1   Mm3
........................................................................ugauccauucgauauggcuuu...............   1     0   Mm3

.................................agccuuaucuuaugugaucaaaug...................................................   3     0   Fm3
......................................................................ugugauccauucgauauggcu.................   10    0   Fm3
......................................................................ugugauccauucgauauggcuuu...............   2     0   Fm3
```
